# Antihyperlipemic and antihypertensive effects of *Spirulina maxima *in an open sample of mexican population: a preliminary report

**DOI:** 10.1186/1476-511X-6-33

**Published:** 2007-11-26

**Authors:** Patricia V Torres-Duran, Aldo Ferreira-Hermosillo, Marco A Juarez-Oropeza

**Affiliations:** 1 Department of Biochemistry, School of Medicine, National Autonomous University of Mexico. P.O. Box 70-159, Mexico, D.F. 04510, Mexico

## Abstract

**Background:**

*Spirulina maxima *is a filamentous cyanobacterium used as food supplement because of its high nutrient contents. It has been experimentally proven, *in vivo *and *in vitro *that posses several pharmacological properties. The purpose of this study was to evaluate the effects of *Spirulina maxima *orally supplied (4.5 g/day, for 6 weeks) to a sample of 36 subjects (16 men and 20 women, with ages between 18–65 years) on serum lipids, glucose, aminotransferases and on blood pressure. The volunteers did not modify their dietary habits or lifestyle during the whole experimental period. From each subject, a sample of blood was drawn in fasting state of 12 hours to determi the plasma concentrations of glucose, triacylglycerols (TAG), total cholesterol (TC), cholesterol associated to high density lipoprotein (HDL-C) and aspartate aminotransferase (AST). Anthropometric measurements including systolic (SYST-P) and diastolic (DIAST-P) blood pressure, height, weight and Body Mass Index (BMI) were also recorded.

**Results:**

Comparing initial and final data, the results showed that there were no significant changes in the values of glucose and AST, but significant differences in TAG, TC, and HDL-C, were observed: TAG 233.7 ± 177.8 vs. 167.7 ± 100.7 mg/dL (p < 0.001), TC 181.7 ± 37.5 vs. 163.5 ± 34.4 mg/dL (p < 0.001), C-HDL 43.5 ± 14.4 vs. 50 ± 18.8 mg/dL (p < 0.01). The univariated analysis showed that the changes in the HDL-C and TC concentrations were dependent on TAG concentration (p = 0.247 and p = 0.108, respectively); nevertheless the calculated values for cholesterol associated to low density lipoprotein (LDL-C) were significantly reduced by the *Spirulina maxima *treatment but independently of the TAG changes. In addition, significant differences were found comparing initial and final SYST-P and DIAST-P blood pressure in both male and female: SYST-P male 121 ± 9 vs. 111 ± 8 mm Hg (p < 0.01), DIAST-P male 85 ± 6.5 vs. 77 ± 9 mm Hg (p < 0.01); SYST-P female 120 ± 9.5 vs. 109 ± 11 mm Hg (p < 0.002), DIAST-P female 85 ± 11 vs. 79 ± 7.5 mm Hg (p < 0.03).

**Conclusion:**

The *Spirulina maxima *showed a hypolipemic effect, especially on the TAG and the LDL-C concentrations but indirectly on TC and HDL-C values. It also reduces systolic and diastolic blood pressure.

## Background

Chronic-degenerative diseases are a growingly health problem all over the world [1]. In Mexico, coronary heart disease and diabetes mellitus are the first and second leading causes of death between general population according to Statistics, Geography and Informatics' National Institute (INEGI) [2]. This has been related with recognized environmental factors as a high caloric diet intake, leak of exercise, tobacco and alcohol consumption and genetic predisposition [3-7]. It is well known that the modification on lipid concentration is a useful approach to decrease cardiovascular mortality through prevention on development of atherosclerotic disease [8-13].

*Spirulina maxima*, a filamentous and unicellular alga is a cyanobacterium belonging to the Oscillatoraceae family that usually grows in the alkaline waters of Africa, Asia, North and South America [14]. *Spirulina *has been used as food additive because of its high content of proteins as well as essential nutrients like carotenoids, vitamins and minerals [15-17]. In addition, previous studies have demonstrated its several biological activities such as inhibit viral replication [15,18,19], prevent anemia [20], decrease genotoxicity induced by drugs [21], prevent fatty liver disease [22-25] and has hypoglycemic [26] and hypolipemic properties [27-30]. It has also been studied its effects on vasomotor responses on aortic rings proposing its antihypertensive activities in experimental models [31-33]. At this point, only few studies have evaluated these effects on human population.

The purpose of this study was to evaluate the effects of *Spirulina maxima*, orally administered, on serum lipids and blood pressure in a Mexican population after six weeks of treatment, as a possible alternative treatment for dyslipidemia and hypertension.

## Results

No changes were observed on AST and glucose values throughout the experimental period (35 ± 18 UI/L and 85 ± 13 mg/dL respectively, results not shown). On the other hand, as shown in Table 1, the initial concentration of lipids did not differ significantly between male (n = 16) and female (n = 20), suggesting their possible association as a single group. Because of the initial triacylglycerol concentrations were 254 ± 173 and 217 ± 184 mg/dL, for male and female groups, respectively, the values suggest a hypertriacylglycerolemic tendency in the studied sample. The other lipid values were in the optimal or limit ranges. In addition, both initial systolic and diastolic blood pressures did not differ significantly between male and female.

**Table 1:** Basal Values in the sample, by gender

	***TAG (mg/dL)***	***TC (mg/dL)***	***HDL-C (mg/dL)***	***LDL-C (mg/dL)***	***SYST- P (mmHg)***	***DIAST-P (mmHg)***
**M**	254 ± 173	177 ± 39	39 ± 13	100 ± 37	121 ± 9	85 ± 6.5
**F**	217 ± 184	186 ± 37	44 ± 15	112 ± 25	120 ± 9.5	85 ± 11
p	0.89	0.647	0.588	0.5	0.586	0.185

Values are mean ± SD. M, male; F, female. TAG, triacylglycerols; TC, total cholesterol; HDL-C, high density lipoprotein-cholesterol; LDL-C, low density lipoprotein-cholesterol. p: significance value, Student's-t test.

At the end of the experimental period there were significant differences in blood lipids after the *Spirulina *treatment (Table 2). Plasma TAG and TC concentrations were the most diminished (p = 0.001, Mann-Whitney and Student's-t tests) but LDL-C concentrations were also decreased (p = 0.01, Student's-t test), and HDL-C values were increased (p = 0.01, paired Mann-Whitney test). However, the univariate analysis showed that the changes on HDL-C and TC concentrations were dependent on TAG concentration (p = 0.247 and p = 0.108, respectively); while LDL-C concentration was independent of TAG values (p = 0.044). As mentioned before, there were no differences between initial and final AST or glucose concentration. Respect on blood pressure (Table 2) it was shown a significant difference between initial and final systolic and diastolic blood pressure records (p < 0.001 SYST-P; p < 0.05 DIAST-P, Tukey's comparison test). Furthermore, a significant decrease on systolic blood pressure was observed since the fourth week of *Spirulina *consumption (initial SYST-P 121 ± 9 *vs*. fourth week SYST-P 114 ± 10 mmHg, p < 0.05; *vs*. fifth week SYST-P 112 ± 8 mmHg, p < 0.01).

**Table 2:** Values of the studied variables, before and after *Spirulina *treatment

***Variable***	***TAG (mg/dL)***	***TC (mg/dL)***	***HDL-C (mg/dL)***	***LDL-C (mg/dL)***	***SYST-P (mmHg)***	***DIAST-P (mmHg)***
**Initial**	234 ± 178	182 ± 37	43 ± 14	103 ± 30	120 ± 9	85 ± 9
**Final**	168 ± 101	163 ± 34	50 ± 19	86 ± 28	109 ± 9	79 ± 8
**p**	0.001^a^	0.001^a^	0.01^a^	0.013^a^	< 0.001^b^	<0.05^b^
Univar. p		0.108	0.247	0.044		

Values are mean ± SD; n = 36; ^a ^Student's t test or ^b ^Tuckey's multiple comparison test; Univar. p = univariated p. p: significance value.

### Dyslipidemia Prevalences

On the other hand, when the effects of *Spirulina *on dyslipidemia prevalences were assessed, the results (Table 3) show that the initial hypercholesterolemia prevalence (TC ≥ 200 mg/dL) was of 27.8%, but it was diminished after the treatment to 13.9%. The most important changes were observed if the higher values of cholesterol (TC ≥ 240 mg/dL) were used for the analysis of hypercholesterolemia, the initial prevalence was 8.3%; and after the *Spirulina *treatment was 0.0%. Initial hypoalphalipoproteinemia prevalence was 27.7% (10/36, cases); and at the end of treatment, prevalence was only 22.0% (8/36 cases). The initial prevalence of hypertriacylglycerolemia in the sample was 41.7% versus a final prevalence of 22.2%. Furthermore, significant differences were found between male and female groups (initial hypertriacylglycerolemia prevalence 62.5% vs. 25.8%, p = 0.026; final prevalence 43.8% vs. 5%, p = 0.008, respectively).

**Table 3:** Dyslipidemia prevalence in the sample (cases)

**Variable**	TAG* (mg/dL)		TC* (mg/dL)		HDL-C* (mg/dL)		LDL-C* (mg/dL)	
	<200	>200	<(200/240)	>(200/240)	<35	>35	<130	>130
	
**Initial (n = 36)**	21	15	26/33	10/3	10	26	27	9
	
**Final (n = 36)**	28	8	31/36	5/0	8	28	33	3

* p < 0.05 initial *vs*. final, chi-squared test

Dyslipidemia prevalences were analyzed in age terciles, with middle age groups of 29.3 ± 5.4 (18–38 years), 43.8 ± 2.7 (39–46 years) and 55.9 ± 5.9 (49–65 years). The results showed that according to the initial values, all of the groups showed hypertriacylglycerolemia cases with predominance (6/36) in the last group (49 to 65 years) whereas most hypercholesterolemic (6 or 3/36, CT >200 or 240 mg/dL, respectively) and hyperbetalipoproteinemic cases (5/36) belong to age group of 39–46 years. After the *Spirulina *treatment, the results showed that the eldest age group (49–65 years) was most responsive to the treatment (p < 0.001). The average change in TAG and TC values after treatment was -20% (-44 to -4, confidence intervals 95%) and -8% (-24 to -2, confidence intervals 95%), respectively. An increase tendency 27% on HDL-C concentration for all groups (5 to 50, confidence intervals of 95%) was observed.

### High Blood pressure prevalence

According JNC 7 blood pressure reference values, high blood pressure prevalence was assessed in total sample before and after treatment with *Spirulina *(Table 4). The results show that the initial Hypertension type 2 prevalence was 14% (5/36 cases), but it was diminished after the treatment to 3% (1/36 case), whereas Hypertension type 1 prevalence diminished from 31% (11/36 cases) to 11% (4/36 cases). Furthermore, an increase on pre-hypertension prevalence from 44% (16/36 cases) to 50% (18/36 cases) and normal blood pressure prevalence from 11% (4/36 cases) to 36% (13/36 cases) were found.

**Table 4:** Initial and Final High Blood Pressure Prevalences* (%)

**BP**	**Normal**	**Prehypertension**	**Hypertension Stage 1**	**Hypertension Stage 2**
**Initial**	11	44	31	14
**Final**	36	50	11	3

BP: blood pressure. * p = 0.01 initial *vs*. final, chi-squared test, n = 36.

Prevalences were analyzed by gender (Table 5). The results showed an initial high prevalence of Stage 1 of Hypertension in both male and female population (30% and 31%, respectively) that was decreased after treatment in both (6% and 15%, respectively) and in the case of stage 2 of hypertension, prevalence in men was decreased from 6% to 0% and in female was reduced from 20% to 5%. The most significant decrement in blood pressure was observed in the youngest group (18 – 38 years; p < 0.001), with an average change in SYST-P and DIAST-P of -8% (-17 to -3, confidence intervals 95%) and -6% (-12 to -1, confidence intervals 95%), respectively.

**Table 5:** High Blood Pressures Prevalences by gender, cases (%)

	**Normal**	**Prehypertension**	**Stage 1 Hypertension**	**Stage 2 Hypertension**
**Male, initial**	1 (6%)	9 (57%)	5 (31%)	1 (6%)
**Male, final**	6 (38%)	9 (56%)	1 (6%)	0 (0%)
**Female, initial**	3 (15%)	7 (35%)	6 (30%)	4 (20%)
**Female, final**	7 (35%)	9 (45%)	3 (15%)	1 (5%)

## Discussion

Previous studies have demonstrated the hypolipemic activity of *Spirulina maxima *in rats with and without toxic substances [27-30]. Using a single intraperitoneal dose of 2 mL/kg carbon tetrachloride (CCl_4_) as an hepatotoxic in order to induce non alcoholic steatohepatitis, it was demostrated that 5% *Spirulina maxima *in diet decrease serum AST, liver TAG and TC in rats. The same pattern was observed in the liver free fatty acids (with an important decrease on unsaturated fatty acids) and thiobarbituric acid reactive substances [24]. These results suggest that *Spirulina *has hepatoprotective properties through decreasing on liver lipid profile and lipoperoxidation products. At this way, it has been demonstrated that *Spirulina maxima *prevents the development of fatty liver induced by simvastatin, ethanol and hypercholesterolemic diet in mice [29].

As previously mentionated, the purpose of this study was to evaluate the effects of *Spirulina *on human lipid metabolism and blood pressure levels. Parikh P et al., studied the effect of *Spirulina *supplementation at 2 g/day doses for two months on blood glucose levels, glycosylated hemoglobin and lipid profile of twenty-five diabetic type 2 subjects [26]. They found a lowering of fasting and postprandial blood glucose levels and in the HbA1c level; this findings contrast with our results, where glucose levels kept stable during all studied period. Respect on lipid profile, the same report demonstrates a reduction on TAG, TC and in the atherogenic indices TC/HDL-C and LDL-C/HDL-C. Samuels R et al., also observed these effects in patients with hyperlipidemic nephrotic syndrome after supplementation with 1 g/day in the same period [34].

In our study, TAG, TC and LDL-C showed a significant reduction with an increase on HDL-C concentration after a study period of six weeks. However, changes on TC and HDL-C were dependent in TAG concentration, meanwhile LDL-C values decreased after treatment in an independent way. Before treatment, a high prevalence of hypercholesterolemia (27.8%, > 200 mg/dL), hypertriacylglycerolemia (41.7%) and hypoalphalipoproteinemia (27.7%) was found; this observation contrast with a Mexican nationwide previous study where hypertriacylglycerolemia was the second most common dyslipidemia (24.3%) after hypoalphalipoproteinemia (46.2% for men and 28.7% for women) [35]; however, the prevalence of hypercholesterolemia was similar to that reported in a more recent study (26.5%) [36]. At this point, after treatment it was observed a significant decrease in all prevalence parameters studied with a major effect observed in the oldest age group (49–65 years). This is very important because a lot of chronic-degenerative disorders related with dyslipidemia, incrementing their prevalence in this age group [1,13,37,38], could be attenuated with *Spirulina *consumption.

With regard to blood pressure, only 11% of studied population had normal levels before treatment, whereas 44% prehypertension, 31% hypertension stage 1 and 14% a stage 2. The hypertension prevalence on Mexican people has shown a clear increasing trend, 13.4% on 2000 survey to 22.7% on 2006 survey, indicating that the present prevalence (45%) could not represent the nationwide values [36]. According the Seventh Report of the Joint National Committee on Prevention, Detection, Evaluation, and Treatment of High Blood Pressure (JNC 7) criteria [39], patients with prehypertension or hypertension in any stage, have to include in their treatment a balanced diet and exercise. Several natural products have been used to help in the treatment of hypertensive patients. Lima-Landman et al., in normal and hypertensive rats demonstrated that an aqueous extract of dried leaves from *Cecropia glaziovii Sneth *had an antihypertensive effect [40]. Ojewole J et al., showed that *Persea americana Mill *aqueous leaf extract (25–800 mg/mL) caused bradycardia, vasorelaxation and hypotension in mammalian experimental models in response to a possibly increased synthesis and release of nitiric oxide [41]. After supplementation with *Spirulina *it was observed 14% hypertension prevalence, 36% patients reached a normal blood pressure whereas patients with hypertension stage 1 or 2 decreased their levels to prehypertension (50%). These changes were observed in men and women, and that youngest group was more blood pressure responsive to *Spirulina *than the other age groups (data not shown).

The action mechanisms of *Spirulina *on lipid metabolism and blood pressure are not well understood yet. Nagakoa S et al., found that a concentrate of *Spirulina platensis *inhibited jejunal cholesterol absorption and ileal bile acid reabsorption, proposing that C-phycocyanin it's the molecule responsible for this effect [42]. Li-Kun H et al., isolated an active component designed as glycolipid H-b2 and they found that this glycolipid inhibits pancreatic lipase activity in a dose-dependent manner, lowering rat plasma TAG levels [43]. Furthermore, they found that phycocyanin inhibits pancreatic lipase at the same way. These effects could explain the hypocholesterolemic and hypotriacylglycerolemic effects seen on *Spirulina maxima*, treated patients, but no studies have been conduced at the moment.

On other hand, a diet supplemented with *Spirulina maxima *has showed to prevent synthesis and release of vasoconstricting metabolites of arachidonic acid induced by fructose and attenuates tension development in response to phenylephrine [33]. Other reports, using an ethanolic extract, demonstrated an increased nitric oxide endothelium synthesis/release, a well known vasodilatation metabolite [31]. At this point, it's well known that blood pressure could be increased in patients that develop vasoconstriction in response to certain metabolites or due to atherosclerotic process [44]. Hypotension observed on patients could be explained on these effects. Hsiao G et al., have proposed that C-phycocyanin inhibits platelet aggregation through inhibition of calcium mobilization and mediation of free radicals released by platelet [45]. These could be supported by a well recognized effect of *Spirulina *mediating several steps on inflammation process that finally reduces atherotrombotic plaque formation. Guan Y et al., have also proposed that high potassium, and low sodium contents of *Spirulina*, have positive effects on blood pressure [46]. We found that supplementation with *Spìrulina *decreases LDL-C and increases HDL-C with a probably beneficial effect on atherotrombotic indices. Finally, our results also supported vasodilatation theory, as seen in a major effect of *Spirulina *in diastolic blood pressure mainly determinated by peripherical vessel resistance.

Actually, statins are recognized as the first-line therapy for cholesterol lowering. In a study conduced by Berne in type 2 diabetic patients, prescription of rosuvastatin (10 mg/day) by 4 weeks decreased significatively LDL-C levels in a mean percentage of 47.6%, TC in 33.6%, TAG in 19.2% and increased HDL-C in 4.4%, and demonstrated more response on lipid profile in comparison with atorvastatin at the same dose [47]. Rangineni V et al., using dried *Eclipta alba *leaf powder (3 g/day) in mild hypertensive subjects, observed a reduction on LDL-C, TC and TAG with mean percentages of 24%, 17% and 14%, respectively [48]. As mentionated before, treatment with *Spirulina maxima*, decreased significatively LDL-C, TC and TAG in a mean percentage of 17%, 8% and 20%, respectively, and increased HDL-C in 27%. These results support that *Spirulina maxima *could be used as an effective supplement on hypertriacilglycerolemic patients.

Respect of blood pressure, dried extract of *Hibiscus sabdariffa*, a widely used herbal medicinal product administrated for 4 weeks, decreased systolic and diastolic blood pressure in 11.58% and 12.21%, respectively [49]. At this way, *Eclipta alba *dried leaf powder reduced mean arterial pressure by 15% [48]. After treatment with *Spirulina maxima*, systolic and diastolic blood pressure decreased in a mean percentage of 8% and 6%, these results suggest that *Spirulina maxima *could be used as a supplement on blood pressure lowering therapy; however, these findings deserve more research in view that our study was performed in an open sample, non-representative and small population.

Finally, security on the use of *Spirulina *was demonstrated with no AST levels elevation trough evaluation period. There were no reports of adverse effects on population studied. Only idiosyncratic adaptative effects as: headache, flatulence, metheorism and increase of intestinal transit (not diarrhea) were reported on the first week of treatment. This is according with other reports where security of oral administration was studied [50-52].

## Conclusion

The present results demonstrate that *Spirulina maxima *has hypolipemic effects, especially on the TAG concentration and the LDL-C but indirectly on TC and HDL-C values and positive effects on lowering blood pressure. We propose that because of these pharmacological properties, *Spirulina *could be used as a dietary supplement on dyslipidemic and hypertensive patients.

## Materials and methods

### Subjects

Sixty five adult volunteers of both genders were invited to participate in the experiment. All of them met the following inclusion criteria: Mexico City inhabitant, age ≥ 18 years old, engagement to complete the treatment, not known cardiovascular disease and not taking at the time of the study drugs known to affect lipid metabolism or blood pressure values, not even oral antidiabetic drugs or insulin. Individuals with Renal Failure or Hepatic Disease were excluded. Only thirty six participants concluded the evaluation period. The final participants were sixteen male and twenty female. The protocol and the aim of the study were fully explained to the subjects, who gave their written consent. The research was carried out in accordance with the Declaration of Helsinki and was approved by the Ethics Committee of the School of Medicine, UNAM, Mexico D.F.

### Study design

It was determined an evaluation period of six weeks in order to observe changes on lipid levels before and after treatment with *Spirulina maxima*. During this time all volunteers were asked not to modify dietary habits or lifestyle to minimize the effects on lipids metabolism. At the beginning of the study and every week, blood samples were taken in the morning after a 12 h fasting and 15 min of rest, using blood collection tubes (BD Vacutainer) in order to observe initial and final levels on glucose, TAG, TC, HDL-C, and weekly AST levels to asses the non-hepatotoxical effect of treatment. Systolic and diastolic blood pressure, height and weight were also registered, and BMI was calculated. *Spirulina maxima *was orally consumed, 4.5 g/day (tablets of 0.5 g, 3 tablets each 8 hours approximately) for six weeks. The *Spirulina *used in this study was purchased from Spiral Spring, Mexico. The valuated composition of the tablet was: protein 57.35%, lipids 5.44 % (ether extract), ash 11.41 %, fiber 0.41 % and carbohydrates 25.33% on dry base analyses.

The prevalence of hyperlipidemia was registered both before and after the experimental period. Hypercholesterolemia was defined as TC levels more than 200 mg/dL or 240 mg/dL, according to Adult Treatment Panel (ATPIII or ATPII) recommendations [53,54]. Hypoalphalipoproteinemia was defined as lower HDL-C values than 35 mg/dl, according to ATPII recommendations. Hypertriacylglycerolemia was defined as higher TAG values than 200 mg/dL, according to ATPII recommendations. Hyperbetalipoproteinemia was defined as higher LDL-C values than 130 mg/dL, according to ATPIII recommendations. Normal blood pressure was defined as values less than 120/80 mmHg for SYST-P and DIAST-P blood pressure, respectively. Prehypertension was defined as values of SYST-P blood pressure of 120–139 mmHg or DIAST-P blood pressure of 80–89 mmHg. Stage 1 of hypertension was defined as values of 140–159 mmHg for SYST-P blood pressure or 90–99 mmHg for DIAST-P blood pressure, meanwhile stage 2 was defined as higher values than 160/100 mmHg for SYST-P and DIAST-P blood pressure respectively, according criteria from The Seventh Report of the Joint National Committee on Prevention, Detection, Evaluation, and Treatment of High Blood Pressure (JNC 7) [39].

### Anthropometric measurements

The height was measured using a rigid stadiometer. Weight of subjects, in light clothing, was measured in a calibrated balance scale. BMI was calculated as the weight (kg) divided by the height (m) squared (kg/m^2^). Blood pressure was measured using a sphygmomanometer by auscultatory method. Anthropometrical details of subjects are shown in Table 6.

**Table 6:** Anthropometrical details of the subjects

Variable	Male	Female
Age (years)	41.5 ± 14.6	44.3 ± 9.6
Height (m)	1.68 ± 0.08	1.55 ± 0.05
Weight(kg)	78.56 ± 11.82*	68.38 ± 10.71
BMI (kg/m^2^)	27.89 ± 2.98	28.50 ± 4.42

Values are mean ± SD. BMI, body mass index. * Significantly different from female by Newman-Keuls multiple comparison test, p < 0.05.

### Laboratory analyses

Blood for plasma analyses was drawn into 100 units sodium heparin (USP) freeze-dried sterile glass tubes (BD Vacutainer) and centrifuged at 4,000 × g for 5 minutes at 4°C. Total cholesterol and TAG concentrations were analyzed by using standard enzymatic procedures (Jas Diagnostics, Inc. Mexico) with a spectrophotometer (Genesys 10 UV, Thermo Electron Corporation, USA); HDL-C concentration was measured after precipitation of apo B-containing lipoproteins with precipitation reactive (Roche, Mexico); LDL-C concentration was determined by using the Friedewald's formula modified by De Long D et al. [55]; AST and glucose were measured with an enzymatic kit (Merck, Mexico) by spectrophotometry methods.

### Statistical analysis

Frequency distribution for variables was determined by Kolmogorov-Smirnov test. Comparisons between groups were done using Student's t test, Mann-Whitney's test or Tukey's multiple comparison test, when appropriate. In order to evaluate dependency of TAG changes, univariated analysis was performed. Significant differences between prevalences were assessed with Chi-squared test using contingency tables. Statistical analyses were performed using SPSS for Windows software (version 10.0). Results are expressed as mean ± S.D.

## Competing interests

The author(s) declare that they have no competing interests.

## Authors' contributions

PVTD participated in the collection, design, analysis and interpretation of data and writing of the manuscript; AFH participated in the collection and analysis of data and performed the statistical analysis and writing of the manuscript; MAJO participated in the design, analysis and interpretation of data and writing of the manuscript. All authors read and approved the final manuscript.
